# A powerful hybrid approach to select top single-nucleotide polymorphisms for genome-wide association study

**DOI:** 10.1186/1471-2156-12-3

**Published:** 2011-01-06

**Authors:** Jian Wang, Sanjay Shete

**Affiliations:** 1 Department of Epidemiology, The University of Texas M. D. Anderson Cancer Center, Houston, TX 77030, USA

## Abstract

**Background:**

Genome-wide association (GWA) study has recently become a powerful approach for detecting genetic variants for common diseases without prior knowledge of the variant's location or function. Generally, in GWA studies, the most significant single-nucleotide polymorphisms (SNPs) associated with top-ranked p values are selected in stage one, with follow-up in stage two. The value of selecting SNPs based on statistically significant p values is obvious. However, when minor allele frequencies (MAFs) are relatively low, less-significant p values can still correspond to higher odds ratios (ORs), which might be more useful for prediction of disease status. Therefore, if SNPs are selected using an approach based only on significant p values, some important genetic variants might be missed. We proposed a hybrid approach for selecting candidate SNPs from the discovery stage of GWA study, based on both p values and ORs, and conducted a simulation study to demonstrate the performance of our approach.

**Results:**

The simulation results showed that our hybrid ranking approach was more powerful than the existing ranked p value approach for identifying relatively less-common SNPs. Meanwhile, the type I error probabilities of the hybrid approach is well-controlled at the end of the second stage of the two-stage GWA study.

**Conclusions:**

In GWA studies, SNPs should be considered for inclusion based not only on ranked p values but also on ranked ORs.

## Background

Genome-wide association (GWA) study has recently become a powerful approach for detecting genetic variants for common diseases without prior knowledge of the variant's location or function [1-4]. Currently, almost all the GWA studies are conducted in two stages: certain numbers (10-50) of the most significant single-nucleotide polymorphisms (SNPs) associated with top-ranked p values are selected in stage one, and follow-up is performed in stage two.

This two-stage approach has been widely used and has successfully identified SNPs with novel susceptibility for different complex diseases such as lung [5-7], prostate [8-11], and breast cancers [12-15]; glioma [16,17]; and type 2 diabetes [18-24]. The published GWA studies showed that in different studies, different numbers of the most significant SNPs were selected at stage one for follow-up. For example, in the GWA study of lung cancer, 10 top-ranked SNPs were selected in stage one [5], whereas in the GWA study of type 2 diabetes, 59 SNPs were selected in stage one [21]. While the p value can indicate when the association of an SNP with a disease is statistically significant, it does not consider the associated odds ratio (OR). The rationale for using OR as a selection criterion is that when minor allele frequencies (MAFs) of causal SNPs are relatively low, much less-significant p values would be observed, even if they could correspond to higher ORs, which might be more useful for prediction of disease status; nevertheless, the less significant p value may limit the inclusion of these significant variants in a replication study. Therefore, selecting a certain number of significant SNPs only based on p values might overlook some important genetic variants that could have an even greater effect on the disease causation.

In this paper, we proposed a hybrid approach for selecting candidate SNPs from stage one of a GWA study that is based on both ranked p values and ranked ORs. Based on simulation studies, the power comparison results showed that our hybrid ranking approach was more powerful than the existing ranked p value approach for identifying relatively less-common SNPs. We performed additional simulation studies to investigate the type I error probabilities of the proposed hybrid approach, and found that the type I errors are well-controlled at the end of second stage of the two-stage GWA study.

## Methods

For the hybrid approach proposed in this paper, ORs are considered in addition to p values when selecting SNPs in stage one. For stage one, we selected a set of SNPs: half were selected based on ranked p values, and the other half were selected based on ranked ORs.

To demonstrate the increased power of our proposed hybrid approach, we performed a simulation study. We assumed two independent disease-causing loci: D_1 _and D_2_. At locus D_1_, we set the MAF at 10%, and at locus D_2_, the MAF was set at 40%. Furthermore, we assumed that there were two marker loci, M_1 _and M_2_, which were associated with two disease-causing loci, D_1 _and D_2_, respectively. MAFs of markers M_1 _and M_2 _were also set at 10% and 40%, respectively. The disease loci and their corresponding marker loci were assumed to be in high linkage disequilibrium (LD, *r*^*2 *^= 0.8). For all the SNPs, we used (0, 1, 2) to denote the three genotypes, where the values corresponded to the number of copies of the deleterious allele. This coding assumed an additive model, but different coding for representing a dominant or recessive model could have also been used. Our proposed hybrid approach was not restricted to an additive model. We defined a categorical random variable, *Y *= (0, 1), to indicate the case-control status: 0 represented individuals in the controls and 1 represented individuals in the cases. We used the logistic model defined below to simulate data.

$$\text{Logit }(P(Y=1))=\beta_{0}+\beta_{1}D_{1}+\beta_{2}D_{2}.$$

For the purpose of our study, we chose different ORs for different loci. At locus D_1_, the OR was set as 1.8 (*β*_1 _= 0.5878), and at locus D_2_, the OR was set as 1.5 (*β*_2 _= 0.4055). *β*_0 _was fixed as a constant 2. Since the markers M_1 _and M_2 _were not directly associated with the disease of interest, they were not included in the logistic model for simulation. The specific parameters for simulation studies are listed in Table 1.

**Table 1:** Parameters for Simulation under Alternative Hypothesis

	Disease Locus 1	Marker 1	Disease Locus 2	Marker 2
	**(D**_**1**_**)**	**(M**_**1**_**)**	**(D**_**2**_**)**	**(M**_**2**_**)**
OR	1.8		1.5	
MAF	10%	10%	40%	40%

OR = odds ratio, MAF = minor allele frequency, LD = linkage disequilibrium.

The LD between the disease loci and the corresponding marker loci is 0.8, and the two disease loci are in linkage equilibrium.

In stage one, we first simulated genotypes for the disease-causing loci D_1 _and D_2 _for each individual. Given the dataset of realizations of SNPs D_1 _and D_2_, we randomly generated a disease status for each individual using the logistic model above. Then, conditioned on data of D_1_, genotypes for marker locus M_1 _were simulated using the *r*^*2 *^value of 0.8. Similarly, genotypes for marker M_2 _were simulated conditioned on data of D_2 _with the same *r*^*2 *^value. Because the markers M_1 _and M_2 _were in high LD with the disease loci D_1 _and D_2, _respectively, they were also associated with the simulated disease. In this way, we simulated a large amount of data for the population of interest and then randomly sampled 1,000 disease-related cases along with 1,000 normal controls from this population. In this study, unless otherwise specified, we employed logistic regression to obtain ORs and used Wald's test to assess significance.

To investigate the power for each SNP to be selected in stage one and followed up in stage two, we generated 1,000,000 replicates of a single SNP under the null hypothesis of no association between the SNP and the disease, each replicate with 1,000 cases and 1,000 controls. To mimic realistic patterns of LD, we applied a forward-time population simulation software program (genomeSIMLA) to generate the 1,000,000 unassociated SNPs [25]. We used information about markers on human chromosome 2, including rs numbers, allele frequencies, recombination fractions, and positions, to seed the initial population. The initial population was then advanced through 1,000 generations of mating to create a pool of individuals. The case and control status for individuals was assigned based on a penetrance function assuming one disease-causal SNP. We then randomly permuted the case-control statuses of individuals to break the association between the SNP and disease of interest. Therefore, all the SNPs simulated were unassociated with the disease of interest. The p values and ORs of all simulated markers were evaluated using PLINK [26]. For the purpose of our simulation, we considered markers with a range of MAFs (10%~40%) that covered the MAFs defined for the disease loci. The p values and ORs obtained under the null hypothesis were used to determine the thresholds for selecting SNPs in stage one. We studied different selections of 10, 20, 30, and 40 SNPs in stage one to follow up on in stage two. Basically, we ranked the 1,000,000 SNPs with respect to p values as well as OR values. If investigators decided to select the top 20 SNPs in stage one using the existing ranked p-value approach, the top 20th-ranked p value out of the 1,000,000 p values would be the threshold for selection, and the SNPs in stage one with more significant p values than the threshold would be forwarded to stage two. Using our proposed hybrid approach, two thresholds for selection were considered. One threshold was the top 10th-ranked p value, and the other was the top 10th-ranked OR value. In order to take into account potential overlapping of the sets of top 10 p-value-based SNPs and top 10 OR-based SNPs, we would first pick SNPs based on p value threshold and then pick additional SNPs based on the OR threshold. The SNPs we selected in stage one either had more significant p values than the p value threshold or had larger OR values than the OR threshold. The reason for selecting 10 OR-based SNPs and 10 p-value-based SNPs was to have the same number (20) of total top SNPs carried over to stage two.

Further, we investigated what percentage of the SNPs could reach a genome-wide threshold for declaring significance. Therefore, for each replicate selected in stage one, we simulated independently another 1,000 cases and 1,000 controls using the same simulation approach and the same parameters. We then employed a joint analysis using fixed indicators for stages one and two, as joint analysis of two stages is efficient and always results in increased power to detect genetic variants [27]. For each pair of corresponding replicates from stages one and two, data from both stages were pooled into one data set. We assumed that the data from different stages were from possibly different sites. Therefore, in order to control for the possible confounding effects of sites, we used (1, 2) to denote the indicator for each stage, with 1 representing stage one and 2 representing stage two. Multivariable logistic regression analysis was applied to the SNP and the stage indicator, and the significance was estimated using Wald's test. All results were based on 1,000 replicates.

We performed additional simulations to examine the type I error probabilities of the proposed hybrid approach under the null hypothesis of no association between SNPs and disease. We assumed that there were two marker loci, M_1 _and M_2_, that were not associated with the disease of interest, with MAFs similar to that used in the power studies. We applied the same software, genomeSIMLA [25], to generate the unassociated SNPs. We initialized the population with small ranges of MAFs. We simulated 10,000,000 replicates each with 1,000 cases and 1,000 controls. The case-control status was assigned at random, independent of the markers, so that markers were unassociated with the disease status. As in the power comparison studies, the same sets of p values and ORs of 1,000,000 replicates of a single SNP under the null hypothesis were employed to determine the thresholds for selecting SNPs in stage one to follow up. Similarly, for each replicate selected in stage one, we independently simulated another replicate using the same simulation approach and same parameters and performed a joint analysis using data from both stages.

## Results

Table 2 lists the resulting medians of negative logarithm to base 10 of the p values and ORs based on 1,000 replicates generated in stage one. The medians of OR estimators for the disease-causing loci D_1 _and D_2 _were 1.8016 and 1.4973, respectively, which had good agreement with the ORs used to generate the data sets (1.8 and 1.5). The results for D_1 _and D_2 _were only reported for comparison purposes. In real GWA studies, the causal SNPs might not be genotyped. Most of the real markers studied are probably in LD with the causal SNPs and, therefore, are associated with the disease of interest, such as the markers M_1 _and M_2 _in our simulation study. In stage two, we assumed that D_1 _and D_2 _were not observed, and therefore, we focused only on the study of two markers. The associated markers generally show lower ORs than those of the actual causal SNPs because of the imperfect LD (*r*^*2 *^= 0.8). Therefore, it is not surprising to see that the medians of the OR estimators of markers M_1 _and M_2 _were smaller than the medians of the corresponding disease loci. As discussed previously, when MAFs of disease-causing SNPs are relatively low, p values would be less significant, even if the corresponding ORs are much higher. This situation applies to the following case. Marker M_1 _(MAF = 10%) was associated with a higher OR (OR = 1.6777) but with a less-significant p value (-Log10 of p value = 4.9597); whereas M_2 _(MAF = 40%) had a lower OR (OR = 1.4323) but a much more significant p value (-Log10 of p value = 7.1542). Therefore, ranking SNPs based only on p values will have less power for marker M_1 _to be included in stage two, even though it has a higher OR and is potentially more useful for making predictions of disease status.

**Table 2:** Medians of Significance and Odds Ratios in Stage One Based on 1,000 Replicates

	Stage One			
**Median**	**D**_**1**_	**M**_**1**_	**D**_**2**_	**M**_**2**_

-Log10 of p value	6.0613	4.9597	8.7299	7.1542
OR	1.8016	1.6777	1.4973	1.4323

OR = odds ratio.

### Power comparisons

We reported the powers for each SNP to be selected in stage one in Table 3. We considered ranges of SNPs selected in stage one (10 to 40). The results in Table 3 are grouped into two panels with respect to two different ranking approaches. For example, if an investigator decided to select 20 SNPs (2^nd ^row in Table 3) in stage one using the existing ranked p-value approach, marker M_1 _would be selected for follow-up in 579 out of 1,000 replicates, and marker M_2 _would be selected in 909 out of 1,000 replicates; power to select marker M_1 _would be 57.9%, and power to select marker M_2 _would be 90.9%. However, using our hybrid approach of selecting top SNPs (10 SNPs based on ranked p values and 10 based on ranked ORs) resulted in powers of 89.9% and 87.0% for selecting markers M_1 _and M_2_, respectively. Compared with the existing ranked p-value approach, our proposed hybrid approach was more powerful for selecting marker M_1_, without losing much power for selecting M_2_, in stage one. Considering the above example, power gain for selecting marker M_1 _was 32.0%, whereas power loss for selecting marker M_2 _was only 3.9%.

**Table 3:** Power to Select Top Single-Nucleotide Polymorphisms in Stage One for Replication Based on 1,000 Replicates

Number of Selections	p-Value-Based Ranking Approach		Hybrid Ranking Approach	
	
	**M**_**1**_	**M**_**2**_	**M**_**1**_	**M**_**2**_
10	52.2%	87.0%	88.9%	84.2%
20	57.9%	90.9%	89.9%	87.0%
30	61.1%	92.4%	90.4%	90.4%
40	62.6%	93.0%	91.2%	90.9%

OR = odds ratio.

MAF was set at 10% for M_1 _and 40% for M_2_.

Even though our approach increased power to select marker M_1 _for follow-up in stage two, we wanted to test what percentage of these SNPs could reach a genome-wide threshold for declaring significance. Therefore, for each replicate selected in stage one, we simulated independently another 1,000 cases and 1,000 controls using the same simulation approach and the same parameters. We then employed a joint analysis using fixed indicators for stages one and two (see Methods section). The power comparisons and medians of ORs for the joint analysis are shown in Table 4. The results are arranged into two panels according to use of the existing p-value-based ranking approach and use of our proposed hybrid ranking approach. The p values and ORs were estimated based on the joint analysis from both stages.

**Table 4:** Median Odds Ratios and Powers in Joint Analysis (Stage One and Stage Two Combined)

Number of Selections	Joint Analysis				
	
		p-Value-Based Ranking Approach		Hybrid Ranking Approach	
	
		**M**_**1**_	**M**_**2**_	**M**_**1**_	**M**_**2**_
10	OR	1.7387	1.4467	1.6866	1.4447
	Power at 1.7 × 10^-7^	51.2%	87.0%	79.4%	84.2%

20	OR	1.7342	1.4418	1.6787	1.4467
	Power at 1.7 × 10^-7^	56.3%	90.9%	80.0%	87.0%

30	OR	1.7343	1.4363	1.6801	1.4398
	Power at 1.7 × 10^-7^	58.9%	92.3%	79.7%	90.4%

40	OR	1.7224	1.4379	1.6761	1.4418
	Power at 1.7 × 10^-7^	60.6%	93.0%	81.2%	90.9%

OR = odds ratio.

As expected, all of the p values from the joint analysis were much more significant than those from stage one. We used a genome-wide threshold p value of 1.7 × 10^-7 ^[27] for declaring the significance of the SNPs. Our proposed hybrid approach for GWA study gained considerable power for marker M_1 _over that of the existing ranked p-value approach. For instance, when the number of selections in stage one was 20, the observed powers were 56.3% and 90.9% for selecting markers M_1 _and M_2_, respectively, using the existing ranked p-value approach; when our proposed hybrid approach was used, however, the observed powers were 80.0% and 87.0% for selecting M_1 _and M_2_, respectively. Even when 40 SNPs were selected for follow-up, we still observed a 20.6% increase in power for selecting marker M_1 _with loss of about 2% power for selecting marker M_2_.

In addition, we also investigated power of selecting both markers loci (M_1 _and M_2_) simultaneously. The power results are reported in Table 5, which are organized into two panels with respect to stage one and joint analysis. In the joint analysis, the powers for selecting both markers were decided by the genome-wide significance as before. The results in Table 5 showed that the hybrid ranking approach has higher power to select both markers M_1 _and M_2_. For example, if 20 SNPs were selected in stage one, the observed powers for selecting both markers were 52.3% and 78.2%, respectively, for using the p-value-based approach and the hybrid approach. In joint analysis, the observed powers for selecting both markers were 50.5% and 70.4% for the two different ranking approaches, respectively, and the proposed hybrid approach gained 19.9% increase in power.

**Table 5:** Power to Select Both SNPs (M_1_ and M_2_) in Stage One and in Joint Analysis (Stage One and Stage Two Combined)

Number of Selection	Stage One		**Joint Analysis (Power at 1.7 **× **10**^**-7**^**)**	
	
	p-Value-Based Ranking Approach	Hybrid Ranking Approach	p-Value-Based Ranking Approach	Hybrid Ranking Approach
10	45.0%	75.0%	43.6%	66.0%
20	52.3%	78.2%	50.5%	70.4%
30	56.0%	81.7%	54.0%	71.6%
40	58.1%	82.8%	55.9%	72.8%

### Type I error estimate

Based on 10,000,000 replicates for each marker locus, we first estimated the percentages of replicates of unassociated SNPs selected for follow-up in stage two. If 10 SNPs were selected in stage one, using the hybrid approach, marker M_1 _would be selected for follow-up in 4,365 of 10,000,000 replicates, and marker M_2 _would be selected in 58 of 10,000,000 replicates. When the standard ranked p-value approach was applied, marker M_1 _would be selected for follow-up in 143 of 10,000,000 replicates and marker M_2 _would be selected in 111 of 10,000,000 replicates. If 20, 30, and 40 SNPs were selected in stage one, using the hybrid approach, marker M_1 _would be selected for follow-up in 5,823, 6,736, and 7,803 replicates, respectively, and marker M_2 _would be selected in 111, 227 and 250 replicates, respectively. Using the standard ranked p-value-based approach, marker M_1 _would be selected for follow-up in 292, 401 and 511 replicates, respectively, and marker M_2 _would be selected in 250, 362 and 468 replicates, respectively. However, importantly, after performing the joint analysis, we found that none of the replicates that have been moved forward to the second stage could satisfy the genome-wide threshold p value of 1.7 × 10^-7 ^for declaring significance, using either our approach or the standard approach. If we increased the threshold p value for declaring significance to 10^-5^, one replicate of marker M_1 _would be significant on the basis of both the hybrid and ranked p-value approaches. Therefore, we can conclude that the type I error rates of the proposed hybrid approach were well controlled at the end of the experiment (stage two).

## Discussion

In this paper, we proposed a hybrid approach to rank and select SNPs in stage one of a GWA study -- ORs are considered in addition to p values. The results from the simulation study show that the hybrid approach has an increased power for identifying the less-common genetic variants. Meanwhile, we concluded through simulation studies that the type I error rates of the hybrid approach are controlled at the end of the experiment.

In our study, we selected half of the candidate SNPs on the basis of ranked p values and the other half on the basis of ranked ORs. In reality, one could select the same or different numbers of candidate SNPs based on ranked p values and ranked ORs. For example, one could select the ranked-p-value-based SNPs using the common GWA study threshold (e.g., p value < 10^-5^) but select the ranked-OR-based SNPs using a less significant threshold (e.g., p value < 10^-3^) for follow-up in stage two. It should be noted that while ranking SNPs based on ORs, both risk and protective effect SNPs should be considered. In our study, we did not have knowledge about whether extremely large or extremely small effect size is more important; therefore, we reversed ORs corresponding to protective effect SNPs before ranking all the ORs (for ranking purposes only). In our simulation studies, we considered a range of MAFs of 10%~40%. A GWA study with 1,000 cases and 1,000 controls has adequate power to detect the SNPs with an MAF of 10% or higher. To detect variants with smaller MAFs, such as less than 5%, a larger sample size will be needed. The impact of the true OR, average LD and MAF on the power of a GWA study to detect susceptibility SNP markers is discussed in Park et al. [28]. Furthermore, these authors also provide sample sizes required for GWA studies to identify associations.

One may argue that using the ranked p values within the rare variants and ranked p values within the common variants to select candidate SNPs would lead to similar results. The obvious deficiency of this approach is the difficulty in defining a boundary for separating rare and common variants. Furthermore, even if one could define a threshold for rare and common variants (say 10%), among the set of rare (or common) variants, there will still be relatively rare variants and relatively common variants. Therefore, when only selecting top SNPs based on the ranked p values within rare variants, one may still be more likely to select the SNPs that are relatively common in the rare variants set, but miss the SNPs that are relatively rare in the rare variants set. For example, if defining SNPs with MAFs < 10% as rare variants and using the ranked p values within rare variants set, one might not be able to capture the SNP with MAF = 3% but OR = 2.5 in stage one (but select the SNP with MAF = 9% but OR = 1.5, for example), even though the OR value represents a very significant association between this SNP and the disease of interest. We had considered such an approach to our simulated data, where we defined SNPs with MAF = 10% as rare variants and SNPs with MAF = 40% as common variants. The powers for selecting both rare and common variants did not increase, however, and were almost identical to those obtained using the standard ranked-p-value approach (data not shown).

**Figure 1:**
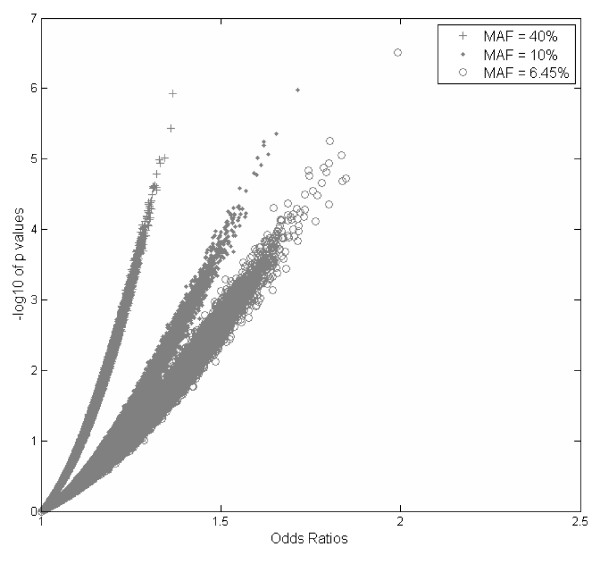
**Odds Ratios versus p Values Based on 1,500,000 Simulated Non-Disease-Related SNPs**.

**Figure 2:**
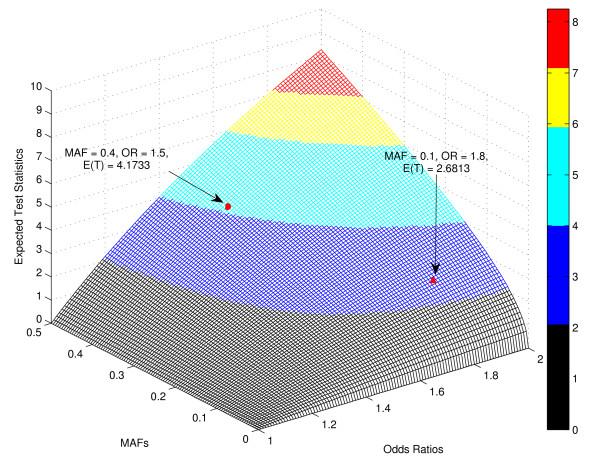
**The Expected Test Statistics E(*T*) under the Alternative Hypothesis as a Function of Minor Allele Frequencies (MAFs, *ρ*) and Odds Ratios (ORs, exp (*β*)) given a Fixed Sample Size *n *= 2000**. The expected test statistics under the alternative hypothesis were estimated using Equation (1), where we used *σ *= 4.6, *n *= 2000, and assumed an additive underlying genetic model. We observed that, when MAF = 0.4 and OR = 1.5, E(*T*) = 4.1733; when MAF = 0.1 and OR = 1.8, E(*T*) = 2.6813.

**Figure 3:**
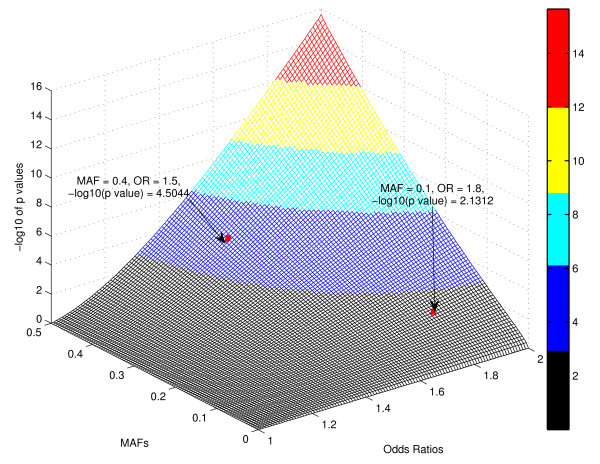
**The Negative Logarithm to Base 10 of p Values under the Alternative Hypothesis as a Function of Minor Allele Frequencies (MAFs, *ρ*) and Odds Ratios (ORs, exp (*β*)) given a Fixed Sample Size *n *= 2000**. The negative logarithm to base 10 of p values is corresponding to the expected test statistics in Figure 2 under the alternative hypothesis. We observed that, when MAF = 0.4 and OR = 1.5, -log10 of p value = 4.5044; when MAF = 0.1 and OR = 1.8, -log10 of p value = 2.1312.

It is well known that large ORs usually correspond to rare variants, and significant p values correspond to common variants, regardless of whether or not the variants are associated with the disease of interest. In our simulation study, we actually found that this is the case, and this can further verify the rationale of our hybrid approach. Figure 1 shows the relationship between p values and ORs based on 1,500,000 null SNPs (no association with disease), where 500,000 SNPs were simulated using MAF = 6.45%, 500,000 SNPs were simulated using MAF = 10%, and 500,000 SNPs were simulated using MAF = 40%. We observed that, at the same p value levels, SNPs with MAF = 6.45% always have the largest ORs; while at the same OR levels, SNPs with MAF = 40% always have the most significant p values. In addition to the simulation study, we also investigated the expected test statistics of association tests, as well as the corresponding p values, under the alternative hypothesis. Figures 2 and 3 show the graphs of the expected test statistics and the p values, respectively, as the functions of ORs and MAFs, given a fixed sample size. The details of the expected test statistics and the assumptions are described in the Appendix. We observed that, for this specific example, when MAF = 0.4 and OR = 1.5, the expected test statistic was 4.1733 and the corresponding negative logarithm to base 10 of p value was 4.5044; when MAF = 0.1 and OR = 1.8, the expected test statistic was 2.6813 and the corresponding negative logarithm to base 10 of p value was 2.1312. Therefore, using the ranked p values approach with a threshold of 10^-4^, the SNP with OR = 1.8 and MAF = 0.1 would not be included in the further analysis, although it has a higher effect size; however, with the use of the hybrid ranking approach, this SNP would have a greater chance of being included in the next stage.

Currently, ranked p value is used as a criterion for selecting common variants, but from Figures 1, 2, and 3, we can conclude that ranked OR should be used as an additional criterion for selecting less-common variants. These two approaches are complementary, and therefore, a hybrid approach using both ranked p value and ranked OR should be more powerful for selecting rare variants. It should be noted that the type I error is controlled in the joint analysis because the SNPs selected in stage one with the use of our hybrid ranking approach are not final, and they have to meet the GWA significance (1.7 × 10^-7^) in joint analysis. The results from the simulation studies confirmed this statement.

## Conclusions

We proposed a hybrid approach for selecting candidate SNPs from the discovery stage of GWA study, based on both p values and ORs, and conducted a simulation study to demonstrate the performance of our approach. The power comparison results show that our hybrid ranking approach is more powerful than the existing ranked p value approach for identifying relatively less-common SNPs. Therefore, GWA studies should consider including SNPs based not only on ranked p values but also on ranked ORs. Furthermore, with the rapid development of sequencing techniques, much denser SNP chips with more low-MAF SNPs may be available in the near future. With these improved technologies, our hybrid ranking approach for selecting top SNPs offers a promising direction for future research in GWA studies.

## Authors' contributions

JW performed the simulation studies and drafted the manuscript. SS conceptualized the study, provided advice and revised the manuscript. All authors read and approved the final manuscript.

## Appendix

Without loss of generality, we considered a simple linear regression model:

$$\begin{array}{cc} y_{i}=\beta x_{i}, & i=1,...n. \end{array}$$

The hypothesis we are interested in testing is *H*_0_: *β *= 0 versus *H*_1_: *β *≠ 0. The estimate $\overset{\wedge }{\beta }$ has the estimate variance $s^{2}/\sum x_{i}^{2}$, where *s *is its estimated standard deviation. Therefore, we can obtain the test statistics *T *as follows:

$$T=\overset{\wedge }{\beta }\times \sqrt{\sum x_{i}{}^{2}}/s.$$

Under the null hypothesis *H*_0_, *T *follows a *t*-distribution with *υ *= *n*-2 degrees of freedom, and if *β *≠ 0, *T *has a noncentral *t*-distribution with *υ *= *n*-2 degrees of freedom and non-centrality parameter $\mu =\beta \sqrt{\sum x_{i}^{2}}/\sigma$[29]. The expected value of the noncentral *t*-distribution test statistics is

(1) $$\begin{array}{cc} E(T)=\mu \sqrt{\upsilon /2}\frac{\Gamma ((\upsilon -1)/2)}{\Gamma (\upsilon /2)}, & \upsilon >1, \end{array}$$

Where Γ (·) is the gamma function. In order to draw the graphs of the expected test statistics and the corresponding p values, we used *σ *= 4.6 and *n *= 2000, and assumed an additive genetic model. Therefore, in the above equation, the degree of freedom is *υ *= *n*-2 = 1998, and the non-centrality parameter is $\mu =\log(OR)\sqrt{n\times (2(\rho^{2}+\rho ))}/4.6$, where *ρ *is the MAF, and OR is odds ratio.
